# Patients Undergoing Subacute Physical Rehabilitation following an Acute Hospital Admission Demonstrated Improvement in Cognitive Functional Task Independence

**DOI:** 10.1155/2014/810418

**Published:** 2014-11-30

**Authors:** Steven M. McPhail, Paul N. Varghese, Suzanne S. Kuys

**Affiliations:** ^1^Centre for Functioning and Health Research, Metro South Health, Buranda, Brisbane, QLD 4102, Australia; ^2^Institute of Health and Biomedical Innovation and School of Public Health and Social Work, Queensland University of Technology, Brisbane, QLD 4059, Australia; ^3^The Princess Alexandra Hospital, Metro South Health, Brisbane, QLD 4102, Australia; ^4^School of Medicine, The University of Queensland, Brisbane, QLD 4006, Australia; ^5^Griffith Health Institute, Griffith University, Gold Coast, QLD 4222, Australia; ^6^The Prince Charles Hospital, Metro North Health, Brisbane, QLD 4032, Australia

## Abstract

*Objective*. This study investigated cognitive functioning among older adults with physical debility not attributable to an acute injury or neurological condition who were receiving subacute inpatient physical rehabilitation.* Design*. A cohort investigation with assessments at admission and discharge.* Setting*. Three geriatric rehabilitation hospital wards.* Participants*. Consecutive rehabilitation admissions (*n* = 814) following acute hospitalization (study criteria excluded orthopaedic, neurological, or amputation admissions).* Intervention*. Usual rehabilitation care.* Measurements*. The Functional Independence Measure (FIM) Cognitive and Motor items.* Results*. A total of 704 (86.5%) participants (mean age = 76.5 years) completed both assessments. Significant improvement in FIM Cognitive items (*Z*-score range 3.93–8.74, all *P* < 0.001) and FIM Cognitive total score (*Z*-score = 9.12, *P* < 0.001) occurred, in addition to improvement in FIM Motor performance. A moderate positive correlation existed between change in Motor and Cognitive scores (Spearman's rho = 0.41). Generalized linear modelling indicated that better cognition at admission (coefficient = 0.398, *P* < 0.001) and younger age (coefficient = −0.280, *P* < 0.001) were predictive of improvement in Motor performance. Younger age (coefficient = −0.049, *P* < 0.001) was predictive of improvement in FIM Cognitive score.* Conclusions*. Improvement in cognitive functioning was observed in addition to motor function improvement among this population. Causal links cannot be drawn without further research.

## 1. Introduction

Hospitalized older adults often experience a decline in physical functioning and mobility in the lead-up to (or during) an acute hospital admission [1, 2]. This loss of functional independence concurrent with hospital admissions may place older adults at risk of undesirable outcomes [3, 4], including potential discharge from hospital to a residential aged care facility rather than returning to their previous community accommodation [2]. Decline in physical function and mobility associated with aging, illness, and reduced physical activity in hospital may occur quite rapidly but require a lengthier period of rehabilitation to recover [5].

During acute illness and hospitalization, older adults may also experience a decline or fluctuation in their Cognitive functioning [4, 6]. Deterioration in Cognitive functioning observed among hospitalized older adults may be permanent, long lasting, or short lived depending on the aetiology, natural disease progression, and clinical management [6, 7]. The presence of reduced Cognitive functioning while in hospital may influence patients' length of stay, participation in decision making with regard to treatment and planning for discharge from hospital, and their ability to govern their own affairs [8, 9]. This may include decision making with regard to whether (or not) they will be able to safely return to their prior community living accommodation. Previous research has indicated that hospitalized older adults with Cognitive impairment also have a greater risk of adverse events while in hospital [4], particularly in-hospital falls which may result in further loss of functional abilities and a greater risk of requiring long-term residential aged care [4, 10, 11].

Prior research investigating Cognitive and physical functioning among older adults in rehabilitation settings has frequently focused on common clinical presentations, with patients recovering from hip fractures or stroke among the most commonly investigated clinical groups in this regard [5, 12–16]. However, older patients who are unable to be safely discharged home due to physical debility, but whose primary reason for admission is attributable to something other than orthopaedic or neurological conditions, are worthy of investigation in their own right [2, 5]. Empirical research among this population to better understand changes in physical and Cognitive functioning, including the effect of age and impaired cognition on potential for improvement, is emerging as an important area of research as societal populations continue to age internationally.

Older adults who have overcome the acute phase of their hospitalization but are not able to carry out functional tasks with the level of independence required to return to their previous accommodation may be considered candidates for subacute inpatient rehabilitation [5]. Patients transferred to specialized subacute geriatric rehabilitation wards receive therapies from multiple disciplines while receiving specialized geriatric medical and nursing care [5]. This typically includes physical and occupational therapies to help maximize functioning in preparation for discharge from hospital. In this context, patients also undertake multidisciplinary assessments of their abilities to complete functional tasks required for daily living; the Functional Independence Measure [17, 18] (including both Cognitive (FIM Cognitive) and Motor (FIM Motor) performance items) is perhaps the most frequently used measure for this purpose in clinical geriatric rehabilitation settings [5, 19]. It is intended that a period of inpatient rehabilitation will give patients experiencing physical debility the best chance of avoiding a need to be discharged to long-term residential aged care and improve their discharge health-related quality of life [3, 5, 20]. However, with increasing demand for beds in specialized geriatric rehabilitation units, it is likely that demand will exceed supply at some facilities. In this regard, it is useful to understand factors that may contribute to patients' potential for benefitting from subacute inpatient rehabilitation.

Previous studies have demonstrated that patients with or without reduced Cognitive functioning on admission to subacute inpatient rehabilitation have considerable potential to improve their physical functioning and quality of life [5, 20–22]. Although, at least, one study among hip fracture patients [12] and another among stroke patients [13] receiving rehabilitation has indicated that better Cognitive functioning at admission (as measured on the FIM Cognitive score) may be predictive of a better outcome from rehabilitation. A range of questions pertaining to the Cognitive functioning of older adults receiving physical rehabilitation interventions for debility following an acute hospitalization have not yet been examined among a sample of older adults not dominated by orthopaedic or neurological conditions as the primary reasons for hospital admission.

This investigation had four aims regarding patients receiving subacute inpatient rehabilitation with debility following an acute hospital admission. The first aim was to summarize the functional independence levels of this clinical group at admission and discharge from subacute inpatient rehabilitation, including the proportion of patients who scored near the upper ceiling of the FIM Cognitive and FIM Motor scores at admission and discharge. The second aim was to describe the levels of functional independence across the individual FIM Cognitive assessment items (at admission and discharge). The third aim was to test whether improvement in FIM Cognitive items had occurred at discharge (in comparison to admission). The fourth aim was to investigate (among patients who were not already near the ceiling of the FIM Cognitive or FIM Motor scoring at admission) (a) the association between change in FIM Cognitive score and change in FIM Motor score over the rehabilitation admission, (b) whether admission FIM Cognitive score (and patient age) was predictive of change in FIM Motor score over the duration of inpatient rehabilitation, and (c) whether admission FIM Motor score (and patient age) was predictive of change in FIM Cognitive score over the duration of inpatient rehabilitation.

## 2. Method

### 2.1. Design

A longitudinal cohort investigation with two assessment points (admission and discharge from subacute inpatient rehabilitation) was undertaken.

### 2.2. Participants and Setting

Participants included patients (*n* = 814) receiving rehabilitative therapies for physical debility associated with an illness or event requiring acute hospitalization and subsequent admission to one of three participating subacute geriatric assessment and rehabilitation hospital wards in Brisbane, Australia. Three criteria were used to exclude rehabilitation patients that were likely to systematically differ from the target sample due to their primary reason for admission. These three criteria were patients whose primary reason for admission was either a neurological condition (e.g., stroke or traumatic brain injury), an orthopaedic condition (requiring surgery, joint immobilization, prescribed movement limitations, or weight-bearing limitations), or a limb amputation (requiring prosthesis specific mobility testing and training). The overarching rationale for these exclusions was that the functional independence (or response to rehabilitative interventions) of individuals in these other common clinical groups may not be consistent with a population of deconditioned hospitalized older adults with physical debility.

Patients in the target sample admitted to the participating subacute rehabilitation wards had typically experienced decline in their physical functioning, particularly their mobility, either in the lead-up to or during their acute hospitalization. Patients in these wards receive interventions from multiple disciplines during rehabilitation designed to optimize their functional abilities at discharge from hospital and thereafter.

### 2.3. Outcomes

Age in years, gender, and primary reason for hospital admission were collected as demographic and clinical variables to describe the sample. The primary outcome of interest was functional independence in tasks dependent on cognition, as assessed by the FIM Cognitive items. Functional independence in tasks dependent on physical functioning, as assessed by the FIM Motor score, was also of interest for addressing Aims 1 and 4.

The FIM is the most frequently reported formal assessment of Cognitive and Motor performance during activities required for daily living among older adults receiving rehabilitative interventions [19]. The instrument includes five Cognitive items and 13 Motor items [17, 18]. Each item is assigned a score from one to seven based on the level of independence with which the subject is able to complete a functional task. An items score of seven indicates the participant is able to complete the task with complete independence. Lower item scores indicate increasing dependence on a helper to complete the task. The maximum FIM Cognitive and Motor score totals are 35 and 91, respectively. The minimal clinically important difference for the Cognitive and Motor scores has previously been estimated at 3 and 17 points, respectively [23]. The FIM Cognitive and Motor items have favourable evidence supporting their reliability [24, 25], validity [17, 26], and responsiveness [27] among older adult and rehabilitation populations. However, ceiling effects have been reported among some rehabilitation patients, particularly in the Cognitive items [27].

### 2.4. Procedure

Patients admitted to the participating hospital wards were assessed by members of the multidisciplinary team within 72 hours of admission. This included completion of the FIM items by each participant's occupational therapist, with input from other members of the multidisciplinary team as necessary. Patients' age, gender, and primary reason for admission were also recorded at the time of rehabilitation admission. All patients received their usual care during this investigation. Patients were assessed again within 72 hours prior to their discharge. This assessment included completion of the FIM items by the participant's occupational therapists following the same procedure as the admission assessment.

### 2.5. Ethical Considerations

This investigation was reviewed and approved by the Metro South Human Research Ethics Committee who granted a waiver of individual consent. Key considerations included that all patients received their usual care and were not required to complete any additional tasks as part of this research investigation (assessments were routine) and that participant privacy and confidentiality was maintained through the removal of personal identifying information. The benefit of this waiver was that it permitted a sample of true consecutive admissions meeting the study criteria. The investigators considered this important to avoid potential selection bias whereby patients dependent on third parties for Cognitive tasks may have otherwise been less likely to be represented in the study sample.

### 2.6. Analysis

**Figure 1:**
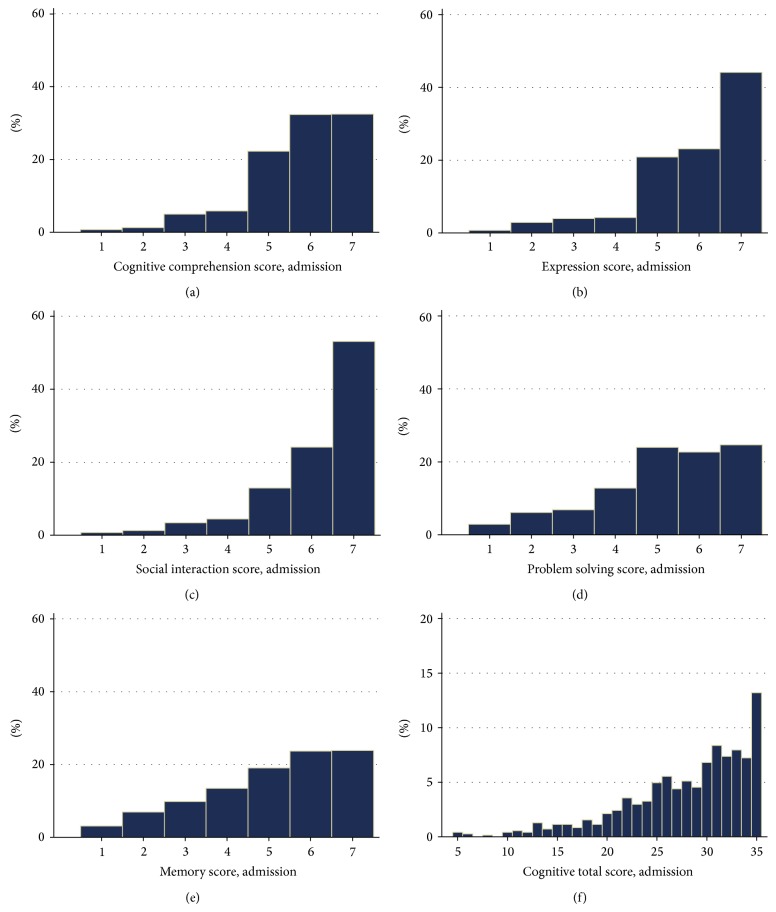
Frequency of Functional Independence Measure Cognitive items scores ((a) to (e)) and total Cognitive score (f) at admission assessment.

**Figure 2:**
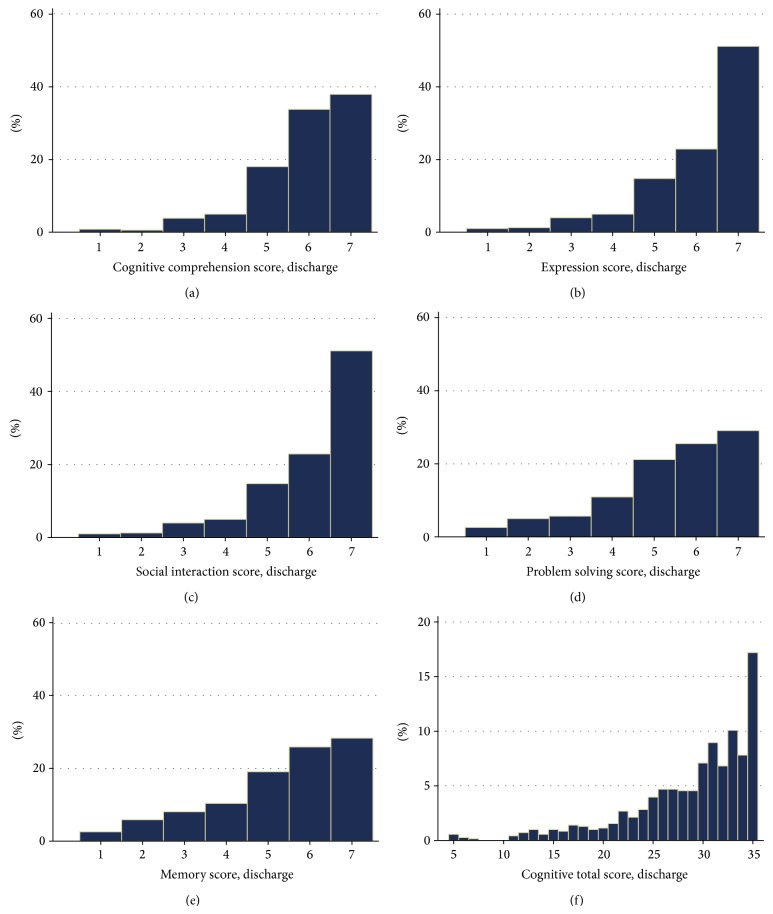
Frequency of Functional Independence Measure Cognitive items scores ((a) to (e)) and total Cognitive score (f) at discharge assessment.

Analyses were performed using STATA IC (Version 11.1). Conventional descriptive statistics (mean, standard deviation, number, and percentage) were used to describe the demographic and clinical characteristics of the sample (Table 1). To address Aim 1, median and interquartile range were used to summarize the participants' FIM Motor and Cognition scores at admission and discharge from rehabilitation. Additionally, the number and percentage of patients assigned scores at the maximum (Cognitive = 35/35, Motor = 91/91) or near the ceiling of the instrument (at maximum or less than a minimally clinically important difference from the maximum) for the FIM Cognitive (>32/35) and Motor (>74/91) score were reported. To address Aim 2, frequency histograms were used to display the patterns of scores assigned to FIM Cognitive items and the total Cognitive items score at admission (Figure 1) and discharge (Figure 2). To address Aim 3, Wilcoxon signed rank tests were used to examine whether a significant difference existed at discharge versus admission for each of the Cognitive items, as well as the total Cognitive score (Table 2). The number (and percentage) of patients who were assigned a higher, equivalent, or lower independence score for each of the Cognitive tasks was also presented alongside *Z*-scores to assist in interpretation of these findings.

Several analyses of associations with change (discharge-admission) in the FIM Cognition and Motor scores were examined to address Aim 4. To mitigate the risk of an instrument scale ceiling effect acting as an artefact unduly influencing these analyses utilizing change scores, patients who had already scored near the ceiling of this instrument at the admission assessment (and thus for whom clinically meaningful improvement during rehabilitation could not be represented on the scale) were not included in these analyses of associations utilizing change scores. Spearman's correlation coefficient was used to describe the strength of direct association between change in the FIM Cognitive score and change in the total Motor score over the duration of inpatient rehabilitation (Aim 4a). Additionally, generalized linear models were also prepared to include age (as an independent variable in the model) when examining (Aim 4a) the association between the Cognition change score (independent variable) and FIM Motor change score (dependent variable), (Aim 4b) whether Cognitive score at admission (independent variable) was predictive of change in Motor score (dependent variable) over the duration of inpatient rehabilitation, and (Aim 4c) whether Motor score at admission (independent variable) was predictive of change in Cognitive score over the duration of inpatient rehabilitation. Due to potential differences in the properties of the variables included in the generalized linear models, a model fitting exercise was conducted to test the sensitivity of the findings to possible family-link functions. This was undertaken by substituting all potential family-link functions; similar model fits and the same significant associations were present regardless of the family-link function selected. Findings using the Gaussian (family) and identity (link) function are presented. As a conservative approach to account for any potential uncertainty in the nature of potential distribution for these observed coefficients, 95% confidence intervals for the coefficients were generated using bootstrap resampling (2000 replications, bias corrected and accelerated to adjust for any potential bias or skewness in the bootstrap distribution; Table 3).

## 3. Results

A total of *n* = 51 (6.3%) patients passed away prior to discharge, and *n* = 59 (7.2%) did not have either the admission or the discharge assessment completed within 72 hours and were excluded from analyses. The remaining *n* = 704 (86.5%) had both assessments completed and were included in analyses. Clinical and demographic characteristics of these participants are displayed in Table 1. The mean (standard deviation) age of these patients was 76.5 (12.2) years; 352 (50.0%) were female.

Participants' FIM Cognitive scores were slightly higher at discharge (median = 31, interquartile range = 26 to 33) than admission (median = 30, interquartile range = 25 to 33). A higher proportion of patients achieved the maximum Cognitive score at discharge (*n* = 121, 17.2%) than admission (*n* = 93, 13.2%). Similarly, a higher proportion of patients achieved a score near the FIM Cognitive ceiling (less than one minimal clinically important difference from the maximum, >32/35) at discharge (*n* = 274, 35.1%) than admission (*n* = 200, 28.4%). Participants' FIM Motor scores were higher at discharge (median = 76, interquartile range = 64 to 83) than admission (median = 55, interquartile range = 41 to 66). A higher proportion of patients achieved the maximum Motor score at discharge (*n* = 19, 2.7%) than admission (*n* = 2, <0.1%). A higher proportion of patients achieved a score near the FIM Motor ceiling (less than one minimal clinically important difference from the maximum, >74/91) at discharge (*n* = 389, 55.3%) than admission (*n* = 74, 10.5%). The remainder of patients who did not score near the instrument ceiling (*n* = 630, >89.5% for FIM Motor, and *n* = 504, 71.6% for FIM Cognitive) had (at least theoretical) potential to improve by a clinically meaningful margin on these scales. This data indicated that patients in this sample admitted for physical rehabilitation not only required a high degree of assistance with functional tasks dependent on their Motor performance at their admission assessment, but assistance was also frequently required with functional tasks dependent on their cognition.

The patterns of scores assigned to FIM Cognitive items and the total Cognitive score are displayed for admission (Figure 1) and discharge (Figure 2) assessments. Few patients were assigned scores lower than 4 out of 7 for the Cognitive tasks at admission (Figures 1(a)–1(e)), although less than half achieved complete independence (score = 7) on any item. Less than 14% (score of 35, Figure 1(f)) were able to complete all five Cognitive items with complete independence at admission. Some variation in the levels of independence achieved across the five Cognitive tasks at admission and discharge was evident, although similar patterns existed within the same item at admission and discharge (Figure 2).

The significance of a trend towards improvement over the duration of subacute inpatient rehabilitation was confirmed for Cognitive items (all *P* < 0.001, *Z*-scores displayed in Table 2) as well as the total Cognitive score (*P* < 0.001, *Z* = 9.12). The proportion of patients who were assigned a higher (more independent), equivalent, or lower score (less independent) at discharge than at admission is displayed in Table 3. These data indicated that a large proportion of patients either improved (*n* = 243, 34.5%) or maintained (*n* = 382, 54.3%) their previous scores and fewer patients demonstrated a reduction in independence.

The examination of association between Motor change score and Cognitive change score indicated that a moderate positive association was present (Spearman's rho = 0.41 (95% CI 0.34–0.49), *P* < 0.001). Each of the generalized linear models produced significant findings (Table 3). In summary, younger age at admission was associated with positive change (improvement) in Motor and Cognitive scores, although the coefficient for age was close to zero in each model (indicating that younger age only had a small effect on the magnitude of improvement in functioning). A better admission Cognitive score had predictive value for improvement in Motor score over the rehabilitation admission.

## 4. Discussion

This investigation successfully addressed each of the research aims. Findings indicated that many older patients admitted for subacute rehabilitation to overcome physical debility following an acute hospital also lack independence in completing functional tasks dependent on their cognition. Over the course of their subacute rehabilitation admission these patients may have not only improved their ability to complete functional tasks dependent on their Motor performance, but a significant improvement in functional tasks dependent on their cognition was also observed. The statistically significant change in median FIM Cognitive score from admission (30 out of 35) to discharge (31 out of 35) for the entire sample may at first glance be considered to be less than a minimal clinically important difference. This highlights the importance of considering this change in median FIM Cognitive score in the context of the proportion of patients who demonstrated improvement on their FIM Cognitive score (only 34.5% of the sample) and the proportion of patients who had already scored near the ceiling of the FIM Cognitive scale at admission (28.4% of the sample). The implication is that genuine improvement in FIM Cognitive performance was observed among a minority of patients in the sample, but this change was sufficient to conclude it was not due to chance. It was also noteworthy that improvement in performance completing FIM Motor items had a moderate association with improvement in performance completing FIM Cognitive items.

These findings are encouraging for patients and health professionals who deliver interventions targeted at optimizing patients' functional independence in order to maximize their quality of life and ability to safely return to community living at discharge. While improvement in Motor performance was to be expected, an associated improvement in independence with completing functional tasks dependent on cognition was an additional benefit. It was also noteworthy that (older) age was significantly associated with (less) improvement in independence with physical or Cognitive functional tasks among this sample. However, the magnitude of this influence (coefficients presented in Table 3) did not indicate that older age posed much of a disadvantage among this clinical group admitted for subacute inpatient rehabilitation.

While the authors are not aware of any comparable studies among this population, the findings from this investigation have consistency with previous investigations among other common clinical groups. For example, a significant association between age and improvement in physical functioning has been reported among older adults receiving rehabilitation following orthopaedic injuries [15, 16]. Similarly, better cognition at admission has previously been shown to have some predictive value for better rehabilitation outcomes following stroke [13].

Findings from the present study are welcome but must be interpreted within the context of this nonneurological or nonorthopaedic patient group, the subacute rehabilitation setting, and the study design. One likely explanation of the observed finding is that this subacute rehabilitation approach that primarily focused on improving physical functioning among this clinical group also improved performance on the FIM Cognitive items among some patients. However, causality from physical interventions cannot be directly extrapolated from this longitudinal cohort investigation. It is plausible that some natural recovery may have occurred whereby Cognitive functioning may have improved over time due to the absence of an acute illness that may have had a residual effect on Cognitive functioning at the admission assessment, but not the discharge assessment.

Consideration of the potential role of delirium in this study is warranted. Prevalence estimates for delirium during acute hospitalisation vary widely but may be as high as 56% [28]. However, it is noteworthy that patients are not typically admitted to the subacute rehabilitation wards participating in this study until after resolution of delirium observed during the acute phase of a patient admission has occurred. A new episode of delirium can occur during a subacute rehabilitation admission, but patients do not routinely complete discharge assessments while still experiencing acute delirium, as they are not typically discharged from hospital while being affected by delirium [29]. Therefore the authors consider it unlikely that improvement in FIM Cognitive performance at the rehabilitation discharge assessment in comparison to rehabilitation admission assessment could be primarily attributable to resolution of delirium.

Another consideration when interpreting these findings is the nature of multidisciplinary rehabilitation provided to patients as well as the content of the FIM Cognitive items. In this setting, improvement in Cognitive item scores should not necessarily be considered equivalent to improvement in neurocognition. For example, time spent participating in multifaceted allied health interventions, group therapy sessions, or other ward-based interactions may have led to greater exposure to social interactions than that which patients may have otherwise recently experienced prior to their admission assessment. Greater participation in these types of interactions could potentially contribute to an improved score on the social interaction item without any change in patients' neurocognition. Although it is interesting to note that moderate to strong associations between the FIM Cognitive score and Mini-Mental State Examination have previously been reported among other clinical groups receiving rehabilitative interventions [13, 30], it is also known that low mood and depression is prevalent among this clinical population [31]. It is possible that improvement in physical health may be associated with improved mood and a reduction in depression which may subsequently be reflected in improved FIM Cognitive item scores.

There are several factors that may limit the ability to extrapolate findings from this investigation to dissimilar populations. The investigation was conducted among older adults who had been referred to and accepted for subacute inpatient rehabilitation. Findings from this investigation may not be applicable to older adults recovering from an acute hospital admission, but who were not admitted for subacute inpatient rehabilitation. For example, patients with moderate or severe dementia who were not considered by their treating clinical teams to be likely to benefit from subacute rehabilitation would not be present in this sample; they would not have been admitted to the participating hospital wards. The presence of a specific Cognitive diagnosis (such as dementia) was not specifically captured in this study, and this could be considered a limitation of the study design. Similarly, the presence (or absence) of delirium in the acute phase of patient admissions was also not able to be accurately determined for the purpose of this study and should be considered a limitation of the study design. Additionally, this investigation was undertaken in a single geographical region in an industrialized nation. Findings from this investigation may not be applicable to dissimilar societies or clinical populations. Furthermore, the FIM was used in this investigation. Other measures of functional independence, cognition, or Motor performance may not have yielded comparable findings.

This investigation has provided empirical data to support or justify future lines of research enquiry. First, an investigation of the effect of physical rehabilitation interventions on neurocognition amongst hospitalized older adults with debility could prove helpful in illuminating the mechanism by which patients in this clinical population improve their Cognitive functioning during their rehabilitation stay. Future research may also investigate functional independence elsewhere in the continuum of patient care, for example, earlier during an acute admission or through the transition from inpatient rehabilitation to community living.

## Figures and Tables

**Table 1:** Demographic and clinical characteristics of the sample.

Participants with both assessments completed	Total *n* = 704

Mean age (standard deviation)	76.5 (12.2) years
Female	*n* = 352 (50%)
Primary reason for hospital admission category	*n*, (%)
Fall^*^	119 (16.9%)
Cardiac	81 (11.5%)
Pulmonary	73 (10.4%)
Vascular	48 (6.8%)
General medicine admission	189 (26.8%)
Other surgical admissions^*^	71 (10.1%)
Musculoskeletal^*^	28 (4.0%)
Other, not elsewhere classified	95 (13.5%)

^*^No patients with neurological conditions, limb amputations, or orthopaedic conditions (requiring surgery, joint immobilisation, prescribed movement limitations, or weight-bearing limitations) were included in the sample.

**Table 2:** Change in Functional Independence Measure Cognitive items scores between admission and discharge from subacute rehabilitation.

Item	Score at discharge in comparison to admission			Wilcoxon signed rank test	
	Lower, *n* (%)	Equal, *n* (%)	Higher, *n* (%)	*Z*-score	*P* value
Comprehension	40 (5.7%)	538 (76.4%)	126 (17.9%)	6.69	<0.001
Expression	31 (4.4%)	554 (78.7%)	119 (16.9%)	7.12	<0.001
Social interaction	55 (7.8%)	543 (77.1%)	106 (15.1%)	3.93	<0.001
Problem solving	35 (5.0%)	533 (75.7%)	136 (19.3%)	7.67	<0.001
Memory	33 (4.7%)	517 (73.4%)	154 (21.9%)	8.74	<0.001

Total	79 (11.2%)	382 (54.3%)	243 (34.5%)	9.12	<0.001

**Table 3:** Summary of coefficients (and bootstrap generated confidence intervals), *Z*-scores, and *P* values from the generalised linear models utilizing Functional Independence Measure change scores.

Dependent variable (aim addressed)	Independent variables	Observed coefficient	95% confidence intervals		*Z*-score	*P* value
			Lower	Upper		
Motor change (Aim 4a)	Cognition change	1.558	1.098	2.019	6.63	<0.001
	Patient age	−0.217	−0.345	−0.089	−3.33	0.001

Motor change (Aim 4b)	Admission cognition	0.398	0.176	0.619	3.52	<0.001
	Patient age	−0.280	−0.417	−0.144	−4.02	<0.001

Cognition change (Aim 4c)	Admission Motor	0.012	−0.010	0.033	1.07	0.28
	Patient age	−0.049	−0.074	−0.025	−3.79	<0.001
